# Recent advances in cosmetic dentistry: A review

**DOI:** 10.6026/973206300211597

**Published:** 2025-06-30

**Authors:** Sidharth Misra, Zainab Yusufali Motiwala, Fatima Nadeem, Krutika Mahendra Gohil, Aditya Puniyani, Gautami Shetty, Sejal Bipin Waghmare, Abhijeet Manohar Nimbalkar

**Affiliations:** 1 Department of General Surgery, Armed Forces Medical College, Pune, Maharashtra 411040, India; 2 Department of Medicine, Jawaharlal Nehru Medical College, Aligarh Muslim University, Aligarh, Uttar Pradesh 202002, India; 3 Department of Medicine, Hinduhridaysamrat Balasaheb Thackeray Medical College and Dr. Rustom Narsi Cooper Municipal General Hospital, Mumbai, Maharashtra 400056, India; 4 Department of Prosthodontics, Terna Dental College & Hospital, Nerul, Navi Mumbai, Maharashtra 400706, India

**Keywords:** Cosmetic dentistry, orthodontics, dental implants, composite bonding

## Abstract

Cosmetic dentistry focuses on enhancing the appearance of teeth, gums, and bite through a multidisciplinary approach involving surgery, orthodontics, periodontics, and restorative techniques. Therefore, it is of interest to review recent innovations, including digital imaging, composite materials, robotic-assisted procedures, and advanced orthodontic systems. Key advancements were noted in teeth whitening, veneers, implants, bonding, and gum contouring. These developments are set to further improve clinical outcomes, patient satisfaction, and accessibility in cosmetic dental care.

## Background:

Cosmetic dentistry is a branch of dentistry that focuses on improving the appearance of the teeth, gums and bite by improving the smile and its aesthetics. It includes procedures that change the colour, shape, size, alignment, or overall appearance of the smile like teeth whitening, dental bonding, veneers, dental implants and composite bonding. By itself, cosmetic dentistry is not a distinct field or specialty of dentistry. However, considering biological and functional factors, it is one of the objectives of dental therapeutic interventions in all specialized areas, including oral and maxillofacial surgery, prosthodontics, orthodontics, periodontics and preventive and restorative dentistry [1]. The quest to improve the appearance of the face and teeth dates back to ancient history [2, 3]. Dentistry emerged as a distinct medical field in the 18th century, aided by the ground breaking research of individuals such as Pierre Fauchard. This allowed for the specialized treatment of both functional and aesthetic dental problems. Dental and facial aesthetics is an essential part of modern dentistry. With the development of science and technology and the advancement of interdisciplinary studies, the research of robot technology in the field of dental and facial esthetics is becoming more extensive and its clinical applications are gradually increasing [4]. Cosmetic dental procedures can be categorised as 'non-surgical' and 'surgical' dental procedures. The non-surgical procedures are those that alter tooth colour, shape or position with minimal damage to the underlying tooth structure. It includes teeth whitening, gum contouring, orthodontics, tooth-bleaching and direct adhesive restorations. 'Surgical' cosmetic dental procedures, cause irreversible change to the tooth, soft tissue and/or bony structures [5]. Its components are the dental veneers, crowns, bridges and implants. Advancements in digital technology are revolutionising the future of cosmetic dentistry. The digital patient data is vital in computer-assisted surgery/dynamic surgical navigation, robots performing dental procedures, computer-aided design and manufacturing [CAD/CAM] and in new approaches to one-appointment restorations [6]. Dental lasers contribute significantly to the field of cosmetic dentistry, providing an invaluable resource for clinicians who perform different types of esthetic procedures [7]. Use of 3D imaging and robot associated interventions aids better communication with patients and greater efficiency and accuracy of dental procedures. Therefore, it is of interest to provide an overview and a comprehensive understanding of the recent advances and modern techniques used in cosmetic dentistry. Furthermore, it describes different surgical and non-surgical components extensively used, their applications and impact on patient outcomes.

## Materials and Methods:

We comprehensively reviewed multiple databases to understand the latest advancements that exist in cosmetic dentistry. A literature search was done on PubMed, Scopus, Embase and Google Scholar in October to November 2024. The articles were reviewed for relevant information on latest innovations in various disciplines of cosmetic dentistry. The literature search used the following key terms: advancements, innovations, breakthroughs, cosmetic dentistry. The selected articles were carefully reviewed for relevant information and the findings from these studies were extracted and presented descriptively. This study involved a review of previously published research studies and did not require ethical approval. This study was deemed low risk. However, we adhered to all relevant ethical guidelines. No human or animal subjects were involved and no new data was collected. All reviewed studies were conducted in accordance with the ethical standards of the respective institutions.

## Major categories of cosmetic dentistry:

Cosmetic dentistry encompasses a variety of procedures that focus on improving the appearance of teeth, gum and smile. They include teeth whitening, gum contouring, composite bonding, orthodontics, as well as placement of dental veneers and implants (Figure 1 - see PDF).

## Teeth whitening:

Teeth whitening are a procedure aimed at lightening the color of teeth, primarily to address discoloration caused by intrinsic or extrinsic factors. This is typically achieved through bleaching, which involves the chemical breakdown of chromogenic compounds within the tooth structure. Whitening can be performed professionally in-office or via at-home methods under supervision. In-office treatments are faster due to higher concentrations of bleaching agents like 30-35% hydrogen peroxide, often enhanced by heat activation, though light irradiation has limited additional benefit [8]. At-home options, including strips, gels, rinses and toothpastes, offer convenience but are less potent. A notable advancement is the KTP 532 nm laser, which effectively penetrates dentin, enhancing gel absorption and improving outcomes, particularly in cases of tetracycline-induced staining. Compared to conventional light-activated systems, KTP laser bleaching ensures quicker results, deeper color changes and minimal adverse effects on oral tissues, making it a safer and more efficient option [9].

## Dental veneers:

Dental veneers are thin, custom-fabricated layers placed on the front surface of teeth to enhance aesthetics and provide protection. The procedure involves enamel reduction, shade matching, impression-taking through digital or conventional methods and final bonding using high-strength adhesives. Porcelain veneers, used since the 1980s, offer excellent esthetics and gingival compatibility, although their failure rates range from 14%-33%, primarily due to poor case selection, inadequate bonding, or occlusal issues. CAD/CAM veneers provide a cost-effective and efficient alternative, delivering standardized quality, durability and a broad shade range with outcomes comparable to traditional techniques [10]. Additionally, lithium disilicate veneers known for their strength and minimal thickness (0.4 mm) enable effective reshaping and restoration in a single visit, making them a reliable option for correcting malformed or unesthetic teeth (Figure 2 - see PDF).

## Dental implants:

Implant-supported dental appliances are surgically inserted devices composed mainly of titanium or ceramic and are used as artificial roots that osseointegrate with the jawbone and bear crowns, bridges, dentures, or prostheses. They increase mastication efficiency, conserve neighbouring tooth structures and enhance speech. Recent developments have achieved a 90-95% success rate and robot-assisted systems such as the KUKA arm further enhance outcomes by minimizing angular and vertical placement errors significantly. The systems aid in overcoming the thin margin of failure inherent in conventional methods by facilitating accuracy and implant stability. Despite technology advancement, difficulties still occur in the precise spatial positioning, particularly in those cases with limited bone volume or anatomical complexity [11, 12]. Infection, sinus or nerve compromise, misalignment and uncommon allergic reactions to titanium are common complications (Figure 3 - see PDF).

## Composite bonding:

Composite bonding is a conservative, aesthetic dental restorative technique in which tooth-colored resin is employed to restore minute defects like enamel cracks, diastemas, or surface staining. The resin is shaped and polished close to the tooth structure and shade. Success with the procedure relies heavily on patient cooperation, with refraining from chromogenic drinks and smoking for the first 48 hours. The development of new technologies, based on nanotechnology, has seen the advent of nanocomposites-nanoscale-sized filler composites with improved polish ability wear and fracture resistance [13]. The nanoscale-sized fillers improve bonding to tooth structure, which translates into natural and durable restorations. Self-healing composites have been proposed as a counter to material degradation, with microcapsules releasing resin upon material crack formation to allow self-healing. Self-adhesive composites, or compobonds, also streamline use by packaging adhesive and composite together in a single formula, reducing postop sensitivity and improving mechanical properties by performing as in-situ shock absorbers [14].

## Orthodontics:

Orthodontics involves the diagnosis, prevention and correction of misaligned teeth and jaws, utilizing a variety of devices such as braces, aligners, retainers and expanders to guide tooth movement and improve bite function. The process begins with comprehensive diagnostic imaging including X-rays, impressions and photographs. Orthodontic arch wires form the foundation of treatment, with brackets-made from metal, ceramic, or composites-transmitting force to the teeth [15]. Aesthetic advancements include self-ligating brackets, which reduce friction and improve tooth movement without the need for elastic ties. Innovations like Invisalign utilize 3D imaging to fabricate a series of clear, removable aligners, while thermal-activated and super-elastic arch wires offer shape memory and continuous, gentle force for enhanced comfort. Opti-Flex wires, composed of optical fibers with moisture-resistant layers, deliver both functional efficiency and aesthetic appeal. For accurate teeth control, customised wire configurations are made possible by the Bending Art technology (BAS), an innovative CAD/CAM wire customisation technology [16]. Additionally, SureSmile creates extremely accurate appliances that offer shorter treatment times, more comfort and better clinical results by combining 3D imaging, robotics and computer-aided design (Figure 4 - see PDF).

## Gum contouring:

Gum contouring, or gum reshaping, is a cosmetic dental treatment designed to enhance smile beauty by modifying the gum line. It is often employed to correct problems like gummy smiles, receding gums and to help prevent tooth decay and periodontal disease advancement [17]. Conducted under local anesthesia, the treatment involves either a scalpel or laser, with lasers providing additional precision, less bleeding and quicker healing. Gummy smile, which is generally characterized by more than 3 mm of exposed gingiva when a person smiles, is an important sign, frequently resulting from overgrowth of the gum tissue, hyperactive upper lip, or short upper lip anatomy. Planning for treatment consists of several factors, such as smile line, gingival biotype, lip dynamics and bone contour. Diode lasers have become increasingly popular for laser-assisted gum contouring due to their accuracy, minimally invasive technique and enhanced aesthetic results [18]. For more severe instances of excessive gingival display, lip repositioning surgeries like the superficial split-thickness dissection or minimally invasive muscle repositioning have yielded good results, with a significant reduction in gingival display and excellent patient satisfaction at follow-up.

## Benefits of robotics in cosmetic dentistry:

Robotics has transformed cosmetic dentistry with unprecedented precision, efficiency and customization in procedures like orthodontics, dental implants and gum reshaping and cosmetic restorations. In orthodontics, robotic technology facilitates precise bending of arch wires and bracket placement, greatly reducing the risk of treatment errors [19]. In dental implants, robotic guidance maximizes optimal placement, minimizing risks like nerve damage and angulation errors. In gum contouring, robotics enables precise gingival reshaping for improved aesthetics and symmetry, making restorations such as veneers or composite bonding fit harmoniously with the patient's own anatomy. Robotic delivery of whitening products guarantees even application and consistent, safe results in all dental arches. In addition, robotics simplifies complicated measurements and improves occlusal balance and functional results. In addition to accuracy, robotic systems significantly minimize treatment time, enhance patient comfort and make the long-term cost more effective. New advances in robotic workflows like for Invisalign aligner production, veneer installation and bonding are also further speeding up delivery times and changing patient experiences in cosmetic dentistry [20].

## Challenges and future directions:

Cosmetic dentistry is confronted with significant challenges, such as the high upfront expenses of sophisticated technologies, restricting access for small practices and impacting mass adoption. Educating dental practitioners to perform complicated procedures is time- and resource-consuming, slowing the integration of new techniques. Patient awareness and acceptance are also low, frequently causing misinformation and reluctance, which calls for improved communication and informed consent. Ethical principles and mutual responsibilities of stakeholders are vital to ensure education and cost-effective implementation. The future is bright with the coming of robotics and AI simplifying intricate procedures, improving accuracy and facilitating highly individualized, patient-specific care.

## Conclusion:

Cosmetic dentistry has seen evolution in terms of the tools and technology involved in it. The accurate and effective surgical treatment that is seen in dental implants, veneers and orthodontics specifically with a view to the outcome for the patients is brought forward. With developments in technology, robotic surgery has been increasingly found to be contributing to the modification of the practice of cosmetic dentistry. Future studies are required to determine the long term implications of robotic assisted treatments in the area of cosmetic dentistry.
